# Correction to: Circulating tumor DNA for monitoring classic Hodgkin lymphoma patients: Correlation with FDG‐PET/CT

**DOI:** 10.1002/jha2.1044

**Published:** 2024-11-04

EJHaem. 2024 Feb; 5(1): 70–75.

The authors regret that the Acknowledgments section contains a mistake in the reference to the funding by the “Instituto de Salud Carlos III (ISCIII)” in the published article.

The correct reference is “This work was supported by Instituto de Salud Carlos III (ISCIII), projects PI19/00083 and PI22/00556 (co‐funded by the European Union)”.

The authors would like to apologize for any inconvenience caused.

